# Clinical and genetic analysis of a family with transthyretin amyloid polyneuropathy caused by a *TTR* Lys55Asn mutation

**DOI:** 10.1186/s13023-025-04148-7

**Published:** 2025-12-12

**Authors:** Nannan Qian, Taohua Wei, Yufei Qian, Wenming Yang, Hui Han, Huaizhen Chen, Jun Li

**Affiliations:** 1 Department of Neurology, The First Affiliated Hospital of Anhui University of Chinese Medicine, Hefei, Anhui 230031 China; 2 First Clinical Medical College of Anhui University of Chinese Medicine, Hefei, Anhui 230031 China; 3 School of Acupuncture-Moxibustion and Tuina, Anhui University of Chinese Medicine, Hefei, Anhui 230038 China

**Keywords:** Autonomic dysfunction, Transthyretin-mediated familial amyloid polyneuropathy, Lys55Asn, *TTR* gene mutation, Case report

## Abstract

**Background:**

transthyretin-mediated familial amyloid polyneuropathy (ATTR-PN), caused by *TTR* gene mutations, leads to systemic amyloid deposition and multisystem dysfunction. The c.165G > C (p.Lys55Asn) mutation is a rare variant with limited clinical data. This study investigates a family with this mutation, focusing on genotype-phenotype correlations and clinical challenges.

**Methods:**

We conducted a detailed clinical analysis of a family with ATTR-PN, using whole exome sequencing to identify the transthyretin (*TTR*) mutation. Clinical data from 17 affected individuals were collected, including symptom onset, disease progression, and outcomes. Electromyography and gastric emptying studies were performed to assess peripheral nerve and gastrointestinal function.

**Results:**

The c.165G > C mutation was confirmed in all affected family members, presenting with early-onset gastrointestinal dysfunction and sensorimotor polyneuropathy. The mean age at onset was 39.76 ± 2.77 years, with rapid progression to death (mean age 46.13 ± 2.97 years) due to cachexia from gastrointestinal complications. Genetic anticipation was observed, with earlier onset in successive generations.

**Conclusion:**

The p.Lys55Asn mutation in the *TTR* gene leads to a severe, rapidly progressive ATTR-PN phenotype, characterized by prominent gastrointestinal dysfunction. This study enhances understanding of the clinical spectrum associated with this rare mutation, emphasizing the need for early diagnosis and targeted management strategies.

**Supplementary Information:**

The online version contains supplementary material available at 10.1186/s13023-025-04148-7.

## Background

 Hereditary transthyretin amyloidosis (ATTRv), also referred to as ATTR-PN, is a rare, autosomal dominant, and life-threatening systemic disorder. It is caused by mutations in the *TTR* gene, which result in the production of an unstable TTR protein that misfolds, aggregates, and deposits as amyloid fibrils in multiple tissues and organs [1, 2]. These amyloid deposits lead to progressive, multisystem organ dysfunction, most commonly affecting the peripheral nervous system, autonomic nervous system, heart, gastrointestinal tract, kidneys, and eyes [3, 4].

The clinical manifestations of ATTR-PN are highly heterogeneous, with considerable variability in age at onset, penetrance, patterns of organ involvement, and disease progression. These differences are influenced by the specific TTR mutation as well as other genetic and environmental factors. Over 140 distinct pathogenic mutations have been identified, each potentially associated with a unique clinical phenotype. The most prevalent genotypes include Val30Met (47.6%), Ser77Tyr (10%), Ala97Ser (6.5%), and Phe64Leu (4.4%). Approximately 18% of cases originate from traditionally endemic countries (e.g., Portugal, Japan, and Sweden), while East Asia (including Japan, China, and South Korea) accounts for 37.0% of cases [5].

This report presents a detailed analysis of a family with ATTR-PN caused by a heterozygous c.165G > C (p.Lys55Asn) mutation in the *TTR* gene. The case illustrates a particularly aggressive phenotype characterized by severe gastrointestinal dysfunction and sensorimotor polyneuropathy, resulting in rapid functional deterioration and premature mortality. This analysis integrates the clinical findings with current literature to explore genotype-phenotype correlations, the challenges in managing severe gastrointestinal manifestations, and the evolving therapeutic approaches that are transforming the prognosis for this devastating disease.

## Case report

**Fig. 1:**
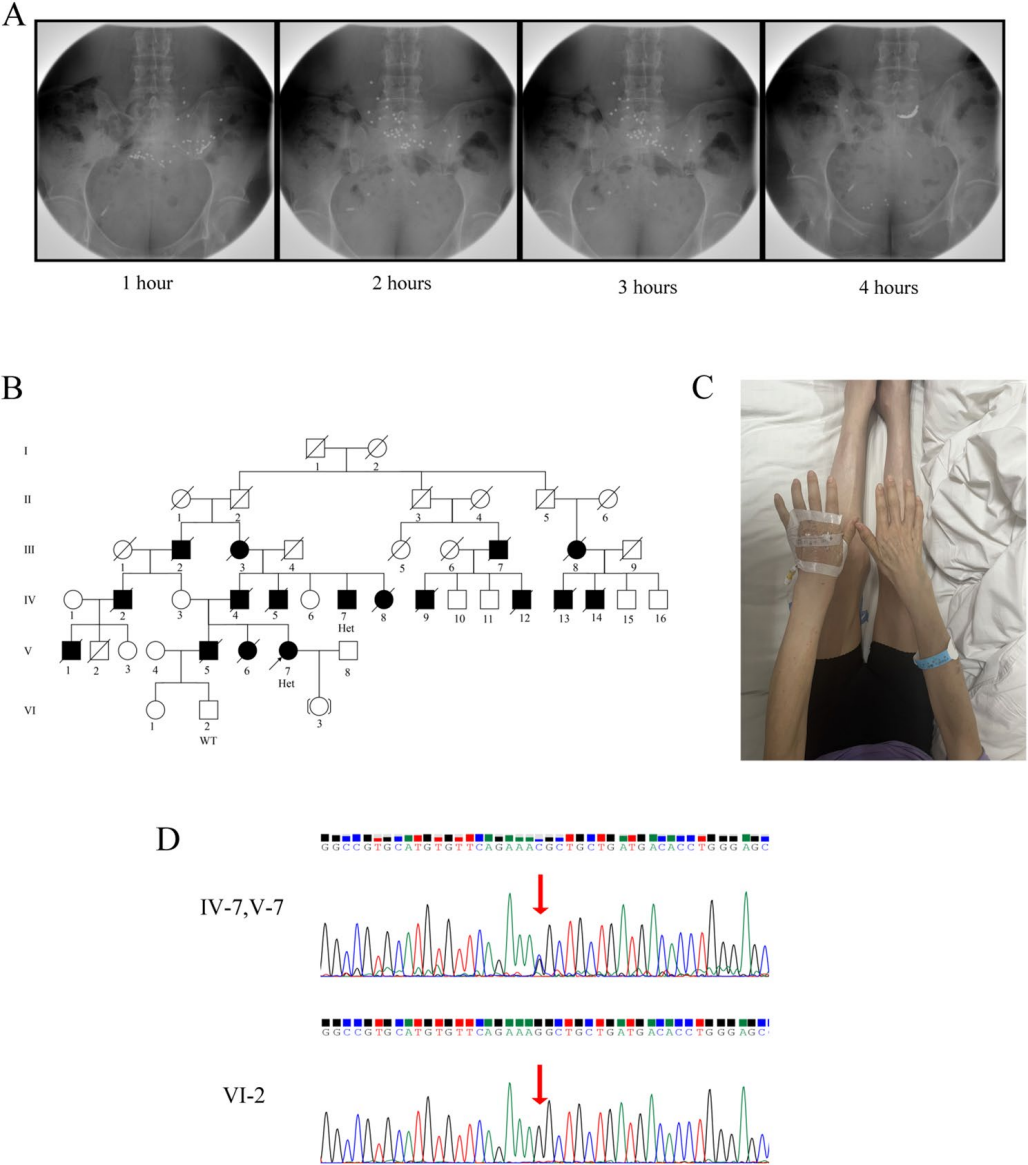
**A**. Gastric emptying test images of patients. **B**. Genetic pedigree of the patient. V-7 is the proband, with a heterozygous genotype; the black filled boxes are the diseased members of the patient’s family. **C**. The patient’s bilateral forearm muscles, thenar muscles, interosseous muscles, tibialis anterior muscles and gastrocnemius muscles were atrophied. **D**. Sanger sequencing images. IV-7 and V-7 showed heterozygous mutations; VI-2 showed no mutation

A 40-year-old female patient presented with a six-year history of recurrent nausea, vomiting, and alternating episodes of diarrhea and constipation. Two years after the onset of gastrointestinal symptoms, she developed progressive numbness and weakness in both lower extremities. The patient exhibited a characteristic steppage gait. Notably, her family history revealed that her father, uncle, cousins, and sister exhibited similar clinical manifestations, and many family members died of cachexia between the ages of 42 and 51 (Fig. 1A; Table 1). Physical examination demonstrated atrophy of the bilateral forearm muscles, thenar muscles, interosseous muscles, tibialis anterior muscles, and gastrocnemius muscles (Fig. 1B). Electromyography showed moderate damage to the motor fibers of the bilateral median and ulnar nerves, as well as severe damage to the sensory and motor fibers of the bilateral common peroneal nerve, tibial nerve, sural nerve, and superficial peroneal nerve (Supplementary Materials). Gastric emptying studies confirmed the presence of gastric emptying dysfunction (Fig. 1C). No abnormalities were detected in the electrocardiogram, echocardiography, renal and bladder ultrasound assessments of residual urine volume, or optical coherence tomography (OCT) of both eyes. Whole exome sequencing identified a heterozygous mutation c.165G > C (p.Lys55Asn) in the *TTR* gene (Fig. 1D), leading to a final diagnosis of ATTR-PN. Following admission, the patient received symptomatic and supportive management. Ondansetron hydrochloride 8 mg was administered intravenously once daily to control nausea and vomiting. Loperamide 2 mg was prescribed orally as needed for the relief of diarrhea. Concurrently, metoclopramide tablets 5 mg and mosapride citrate tablets 5 mg were given orally three times daily to enhance gastrointestinal motility. After one week of treatment, the patient’s gastrointestinal symptoms showed significant improvement and clinical stability was achieved; therefore, the patient was discharged.

**Table 1:** Clinical characteristics of the proband and affected families

	Gender	Age (years)		Survival Duration	Initial complaints	Clinical features
		onset	death			
proband	female	34	-	-	nausea, vomiting	Dysfunction of peripheral nerves Autonomic dysfunction Gastrointestinal symptoms
Ⅲ−2	male	43	50	7	Numbness of upper extremities	Dysfunction of peripheral nerves Ocular dysfunction Autonomic dysfunction Gastrointestinal symptoms
Ⅲ−3	female	42	49	7	Numbness of limbs	Dysfunction of peripheral nerves Autonomic dysfunction Gastrointestinal symptoms
Ⅲ−7	male	45	51	6	cataract	Dysfunction of peripheral nerves Ocular dysfunction Gastrointestinal symptoms
Ⅲ−8	female	41	48	7	decreased vision	Dysfunction of peripheral nerves Ocular dysfunction Gastrointestinal symptoms
Ⅳ−2	male	41	49	8	Carpal tunnel syndrome	Dysfunction of peripheral nerves Autonomic dysfunction Gastrointestinal symptoms
Ⅳ−4	male	40	47	7	cataract	Dysfunction of peripheral nerves Ocular dysfunction Gastrointestinal symptoms
Ⅳ−5	male	39	45	6	Lower extremity weakness	Dysfunction of peripheral nerves Gastrointestinal symptoms
Ⅳ−7	male	43	-	-	cataract	Dysfunction of peripheral nerves Ocular dysfunction
Ⅳ−8	female	41	48	7	Numbness of limbs	Dysfunction of peripheral nerves Autonomic dysfunction Gastrointestinal symptoms
Ⅳ−9	male	40	45	5	Chest tightness	Dysfunction of peripheral nerves Autonomic dysfunction Cardiac dysfunction Gastrointestinal symptoms
Ⅳ−12	male	38	43	5	palpitations	Dysfunction of peripheral nerves Cardiac dysfunction Gastrointestinal symptoms
Ⅳ−13	male	40	46	6	Carpal tunnel syndrome	Dysfunction of peripheral nerves Autonomic dysfunction Gastrointestinal symptoms
Ⅳ−14	male	38	44	6	Numbness of limbs	Dysfunction of peripheral nerves Autonomic dysfunction Cardiac dysfunction Gastrointestinal symptoms
Ⅴ−1	male	37	42	5	palpitations	Dysfunction of peripheral nerves Cardiac dysfunction Gastrointestinal symptoms
Ⅴ−5	female	38	43	5	Chest tightness	Dysfunction of peripheral nerves Cardiac dysfunction Gastrointestinal symptoms
Ⅴ−6	female	36	42	6	Lower extremity weakness	Dysfunction of peripheral nerves Autonomic dysfunction Gastrointestinal symptoms

### Family clinical features

Table 1 presents the clinical characteristics of the proband and 16 affected family members (total *n* = 17, including 11 males and 6 females). The mean age of onset was 39.76 ± 2.77 years (range: 34–45 years), with values of 42.75 ± 1.71 years (range: 41–45 years) in the third generation, 40.00 ± 1.58 years (range: 38–43 years) in the fourth generation, and 36.25 ± 1.71 years (range: 34–38 years) in the fifth generation. A statistically significant difference in age of onset across generations was observed (*P* < 0.01), indicating a trend toward earlier onset in successive generations. The mean age at death was 46.13 ± 2.97 years (range 42–51), and the mean disease duration from onset to death was 6.20 ± 0.94 years (range 5–8). The initial symptoms included numbness in the lower and/or upper limbs (4 cases), lower limb weakness (2 cases), nausea and vomiting (1 case), cataracts (4 cases), carpal tunnel syndrome (2 cases), and chest tightness or palpitations (4 cases). All patients exhibited varying degrees of dysfunction of peripheral nerves and gastrointestinal symptoms, and all deceased patients succumbed to cachexia resulting from gastrointestinal complications. Additionally, during the disease course, 9 patients developed autonomic dysfunction, 6 patients developed ocular dysfunction, and 4 patients experienced cardiac dysfunction.

## Discussion

This study reports a family affected by ATTR-PN, caused by a heterozygous c.165G > C (p.Lys55Asn) mutation in the *TTR* gene. The family exhibits typical autosomal dominant inheritance, with a distinct clinical phenotype characterized by early-onset, progressive gastrointestinal dysfunction and sensorimotor neuropathy, along with notable familial clustering and genetic anticipation. These findings contribute to a deeper understanding of the clinical heterogeneity of ATTR-PN and provide valuable insights into the phenotypic spectrum associated with the rare *TTR* c.165G > C mutation.

ATTR-PN is a rare, autosomal dominant inherited fatal disorder caused by mutations in the TTR gene. These mutations lead to structural instability of the TTR protein tetramer, resulting in misfolding and systemic deposition in multiple organ tissues, predominantly affecting the peripheral nervous system [6, 7]. Previous studies indicate a global prevalence of 5,000–10,000 patients. The condition was first identified by Corino de Andrade in Portugal [8]. To date, over 140 pathogenic *TTR* mutations have been identified, the majority of which are missense mutations [9]. Distinct mutation types are strongly associated with variable clinical phenotypes, age at onset, and disease progression, demonstrating significant genetic and clinical heterogeneity [6, 10]. The most prevalent mutation is Val30Met, whereas other variants are relatively rare [6, 7, 10].

In this study, the c.165G >C (p.Lys55Asn) mutation, identified through whole-exome sequencing, represents an extremely rare variant of the TTR gene. A systematic review of the existing literature revealed a severe lack of comprehensive reports regarding the clinical features, epidemiological data, and penetrance associated with this specific mutation [2, 8, 9, 11]. To date, only one study on a Chinese ATTR-PN family cohort has briefly mentioned the presence of c.165G >C among five identified *TTR* mutations, yet it did not provide detailed descriptions of the associated clinical phenotype or prevalence data [8]. Therefore, this case report offers a thorough characterization of the clinical syndrome associated with the c.165G >C mutation, contributing valuable evidence for the establishment of a “genotype–phenotype” correlation map for this rare variant. The observed clinical presentation in this family, characterized by marked disease severity and a rapidly progressive course, suggests that the p.Lys55Asn mutation may significantly compromise the structural stability of the TTR protein and confer a high propensity for amyloid formation.

The clinical manifestations observed in this family exemplify the multi-system involvement characteristic of ATTR-PN, with severe gastrointestinal (GI) dysfunction serving as a core and particularly prominent feature. GI symptoms represent a common and clinically challenging issue in ATTR-PN patients, frequently manifesting prior to or concurrently with neuropathic symptoms, and significantly compromising patients’ nutritional status and quality of life [12, 13]. In this study, the proband initially presented with recurrent nausea, vomiting, and alternating episodes of diarrhea and constipation, with a disease course spanning six years. Objective evidence supporting these GI symptoms was provided by the assessment of gastric emptying function (Fig. 1C). Notably, all deceased family members (*n* = 15) ultimately succumbed to cachexia resulting from GI complications, underscoring the central and life-threatening role of GI dysfunction in this pedigree. The pathogenesis of GI dysfunction in ATTR-PN is thought to be associated with amyloid deposition in the autonomic and enteric nervous systems, leading to neuronal degeneration and functional impairment, which in turn result in gastrointestinal motility disorders [14]. The fatal outcomes observed in this family highlight the critical need for proactive nutritional support and targeted symptomatic management, particularly in patients presenting with prominent GI manifestations [12]. Clinical management remains highly challenging and largely reliant on supportive measures such as dietary modifications, prokinetic agents, and antidiarrheal medications, which often yield suboptimal outcomes [14].

Two years after the onset of gastrointestinal symptoms, the proband developed progressive numbness and weakness in both lower extremities, accompanied by a characteristic steppage gait. Physical examination revealed distal muscle atrophy, and electromyography confirmed extensive sensory and motor nerve fiber impairment, findings that are consistent with the typical peripheral neuropathy features of ATTR-PN [15]. The neuropathy observed in this case is symmetrical and length-dependent, initiating in the distal portions of the lower limbs and progressively extending proximally, which represents one of the hallmark clinical manifestations of the disease. All affected individuals in this family exhibited varying degrees of peripheral nerve dysfunction, further supporting the neurotropic nature of this specific *TTR* mutation. Although the core phenotypic features of this family are largely consistent, notable inter-individual variability is also observed. Among the 17 affected individuals, the initial clinical presentations were heterogeneous, including limb numbness or weakness, nausea and vomiting, cataracts, carpal tunnel syndrome, and palpitations, indicating that early disease manifestations may lack specificity. Furthermore, while cardiac involvement, ocular abnormalities, and autonomic dysfunction are present among family members, the proband in this study showed no significant abnormalities on electrocardiography, echocardiography, or ocular optical coherence tomography. This phenotypic heterogeneity, even within a single pedigree, aligns with the well-established clinical and genetic heterogeneity of ATTR-PN, underscoring the complex and variable nature of the disease [4].

The mean age of onset among family members was 39.76 ± 2.77 years (range: 34–45 years). The age of onset in ATTR-PN exhibits considerable variability and is influenced by multiple factors, including mutation type, geographical origin, and genetic background [16, 17]. The third generation had a mean age of onset of 42.75 ± 1.71 years (range: 41–45 years), the fourth generation 40.00 ± 1.58 years (range: 38–43 years), and the fifth generation 36.25 ± 1.71 years (range: 34–38 years). There was a statistically significant difference in age of onset across generations (*P* < 0.01), demonstrating a progressive trend toward earlier symptom manifestation with each successive generation—consistent with the hallmark feature of genetic anticipation, defined as the phenomenon in autosomal dominant disorders whereby clinical symptoms manifest at an earlier age in subsequent generations. The persistent advancement in onset age observed in this family supports the hypothesis that the p.Lys55Asn mutation may be associated with a more aggressive disease phenotype.

A clear pattern of genetic anticipation is present in this family, a phenomenon well-documented in various hereditary neurodegenerative diseases. In ATTR-PN, genetic anticipation has been extensively reported and thoroughly investigated, particularly in families harboring the Val30Met mutation [18]. From a molecular perspective, several mechanisms may underlie genetic anticipation. First, dynamic instability of the *TTR* gene mutation could lead to earlier aggregation of misfolded proteins; specifically, the p.Lys55Asn variant identified in this case may enhance the propensity for amyloid fibril formation, thereby accelerating pathological deposition in neural and gastrointestinal tissues. Second, aberrant epigenetic regulation—including alterations in DNA methylation patterns or histone modification profiles—may disrupt normal gene expression control, enabling mutant protein levels to reach pathogenic thresholds at younger ages. Furthermore, gene-environment interactions must not be overlooked: descendants may experience synergistic effects between inherited susceptibility and exposure to environmental toxins, metabolic stress, or lifestyle changes, collectively contributing to accelerated target organ damage [16, 19–22]. Notably, the average age of onset in this family is approximately 39.76 years, while the mean age at death is only 46 years, yielding an average disease duration of about 6.2 years. This indicates rapid disease progression and extremely poor prognosis, potentially reflecting an association between genetic anticipation and increased phenotypic severity.

Pathological examination plays a pivotal role in diagnosing and differentiating ATTR-PN. The “gold standard” for diagnosing ATTR-PN involves obtaining direct evidence of amyloid deposition through biopsies from various tissues. Additionally, *TTR* gene testing serves as another critical diagnostic tool [23].

The diagnosis of this disease presents two main challenges. First, the multisystemic nature of amyloid deposition can manifest as peripheral neuropathy (sensory-motor disorders), autonomic dysregulation (gastrointestinal motility disorders), myocardial hypertrophy, and vitreous opacities. These symptoms are often mistaken for chronic inflammatory demyelinating polyneuropathy (CIDP) or idiopathic autonomic dysfunction [23]. Second, approximately 20% of patients lack a clear family history, potentially due to de novo mutations or variable penetrance [24]. In such cases, genetic testing and tissue biopsy become essential for accurate diagnosis. Early identification of these “cryptogenic” cases is crucial for improving patient outcomes.

Diflunisal, a *TTR* stabilizer, is suitable for mild stages of the condition, but has limited efficacy against established amyloid deposits [25]. Gene silencing therapy, specifically Patisiran, can markedly reduce serum mutant *TTR* levels, necessitating early intervention to delay organ damage [26]. Management of gastrointestinal motility requires a combination of prokinetic drugs and probiotics. Furthermore, prevention of cardiac complications requires dynamic monitoring of QT interval changes.

## Conclusion

In summary, through a comprehensive analysis of a family harboring the rare *TTR* c.165G > C (p.Lys55Asn) mutation, this study identifies a lethal clinical syndrome characterized by early-onset, severe gastrointestinal dysfunction, and rapidly progressive sensory-motor neuropathy. The presence of marked genetic anticipation and an extremely poor prognosis in this family underscores the high pathogenicity of the p.Lys55Asn mutation. Our findings not only expand the established genotype-phenotype correlations of the *TTR* gene but also highlight the critical need to enhance clinical awareness of ATTR-PN, facilitate early genetic diagnosis, and develop effective therapeutic strategies for severe complications, particularly gastrointestinal involvement.

## Supplementary Information

Below is the link to the electronic supplementary material.

Supplementary Material 1

## Data Availability

The datasets supporting the conclusions of this study are available from the corresponding author upon reasonable request.
