# Supplementary material for: Clinical and genetic analysis of a family with transthyretin amyloid polyneuropathy caused by a TTR Lys55Asn mutation
Source: Orphanet J Rare Dis. 2025 Dec 12;21:20. doi: 10.1186/s13023-025-04148-7 (PMC12817533; doi:10.1186/s13023-025-04148-7)
Supplement: Supplementary file 1 — Supplementary Material 1 [file 13023_2025_4148_MOESM1_ESM.docx]

Table 1. Nerve Conduction > Motor Conduction (MCV)

| **nerve** | **Stimulation area** | **Recording location** | **Incubation period** | **Time limit ms** | **Amplitude mV** | **Speed m/s** |
| --- | --- | --- | --- | --- | --- | --- |
| Right median nerve | wrist | Abductor pollicis brevis | 4.45 | 5.95 | 2.16 | 41.3 |
|  | elbow | Abductor pollicis brevis | 9.90 | 5.60 | 2.01 |  |
| Left median nerve | wrist | Abductor pollicis brevis | 3.60 | 5.00 | 3.13 | 46.4 |
|  | elbow | Abductor pollicis brevis | 8.45 | 6.95 | 2.32 |  |
| Right ulnar nerve | wrist | abductor digiti minimi | 1.50 | 8.90 | 1.73 | 39.8 |
|  | elbow | abductor digiti minimi | 7.15 | 7.65 | 1.96 |  |
| Left ulnar nerve | wrist | abductor digiti minimi | 2.70 | 9.50 | 1.77 | 43.7 |
|  | elbow | abductor digiti minimi | 7.85 | 17.7 | 1.54 |  |
| Right common peroneal nerve | Anterior ankle | Extensor digitorum brevis | 0.40 | 1.20 | 0.00 | - |
|  | Head of fibula | Extensor digitorum brevis | 0.40 | 1.20 | 0.00 |  |
| Left common peroneal nerve | Anterior ankle | Extensor digitorum brevis | 0.40 | 1.20 | 0.00 | - |
|  | Head of fibula | Extensor digitorum brevis | 0.40 | 1.20 | 0.02 |  |
| Right tibial nerve | Inner ankle | Extensor hallucis muscle | 0.40 | 1.20 | 0.00 | - |
|  | Popliteal fossa | Extensor hallucis muscle | 0.40 | 1.20 | 0.00 |  |
| Left tibial nerve | Inner ankle | Extensor hallucis muscle | 0.40 | 1.20 | 0.04 | - |
|  | Popliteal fossa | Extensor hallucis muscle | 0.40 | 1.20 | 0.01 |  |

Table 2. Nerve Conduction > Sensory Conduction Velocity (SCV)

| **nerve** | **Stimulation area** | **Recording location** | **Incubation period** | **Time limit ms** | **Amplitude uV** | **Speed m/s** |
| --- | --- | --- | --- | --- | --- | --- |
| Right median nerve | Middle finger | wrist | 7.30 | 0.87 | 1.97 | - |
| Left median nerve | Middle finger | wrist | 1.00 | 1.90 | 0.17 | - |
| Right ulnar nerve | little finger | wrist | 1.00 | 1.87 | 0.16 | - |
| Left ulnar nerve | little finger | wrist | 1.00 | 2.33 | 8.99 | - |
| Right radial nerve | thumb | wrist | 5.00 | 0.53 | 0.08 | - |
| Left radial nerve | thumb | wrist | 8.53 | 1.87 | 0.16 | - |
| Right sural nerve | 14cm above the calcaneus | Outer ankle | 3.20 | 0.37 | 0.16 | - |
| Left sural nerve | 14cm above the calcaneus | Outer ankle | 1.60 | 0.53 | 0.11 | - |
| Right superficial peroneal nerve | Peroneus longus | Dorsum of foot | 5.13 | 4.57 | 0.66 | - |
| Left superficial peroneal nerve | Peroneus longus | Dorsum of foot | 12.0 | 0.50 | 0.03 | - |

Table 3. Nerve conduction > H reflex (HR)

| **nerve** | **Stimulation area** | **M lurking ms** | **H lurking ms** | **HM lurking ms** | **M amplitude mV** | **H amplitude mV** | **H/M Amplitude Ratio** | **Distance mm** | **Speed m/s** |
| --- | --- | --- | --- | --- | --- | --- | --- | --- | --- |
| Right tibial nerve | No positive waveform is drawn | | | | | | | | |
| Left tibial nerve |  |  |  |  |  |  |  |  |  |
